# Microbial proteasomes as drug targets

**DOI:** 10.1371/journal.ppat.1010058

**Published:** 2021-12-09

**Authors:** Hao Zhang, Gang Lin

**Affiliations:** Department of Microbiology & Immunology, Weill Cornell Medicine, New York, New York, United States of America; Institut Pasteur, FRANCE

## Abstract

Proteasomes are compartmentalized, ATP-dependent, N-terminal nucleophile hydrolases that play essentials roles in intracellular protein turnover. They are present in all 3 kingdoms. Pharmacological inhibition of proteasomes is detrimental to cell viability. Proteasome inhibitor rugs revolutionize the treatment of multiple myeloma. Proteasomes in pathogenic microbes such as *Mycobacterium tuberculosis* (Mtb), *Plasmodium falciparum* (Pf), and other parasites and worms have been validated as therapeutic targets. Starting with Mtb proteasome, efforts in developing inhibitors selective for microbial proteasomes have made great progress lately. In this review, we describe the strategies and pharmacophores that have been used in developing proteasome inhibitors with potency and selectivity that spare human proteasomes and highlight the development of clinical proteasome inhibitor candidates for treatment of leishmaniasis and Chagas disease. Finally, we discuss the future challenges and therapeutical potentials of the microbial proteasome inhibitors.

## Introduction

**Fig 1:**
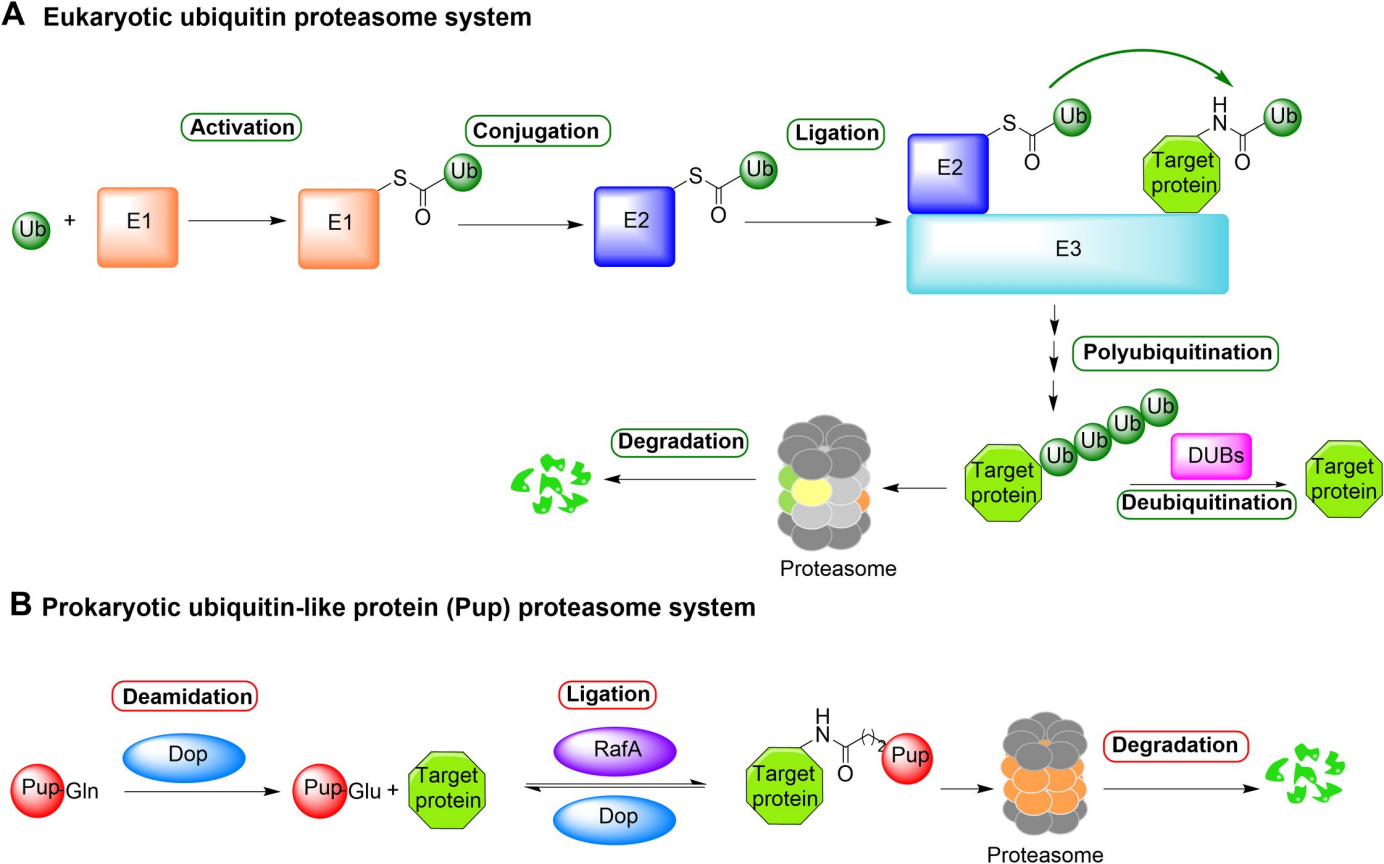
Ubiquitin-proteasome system in eukaryotes and Pup-proteasome system in *Mycobacterium*. DUB, deubiquitinase; Pup, prokaryotic ubiquitin-like protein.

Regulated protein degradation is a pivotal process in all cells [1,2]. The 20S core proteasome is a self-compartmentalized protease in the cytosol and nucleus of eukaryotic cells. In eukaryotes, the ubiquitin proteasome system (UPS) is responsible for the timely degradation of the majority of damaged, unneeded, and regulatory proteins in the proteasome [3,4]. Ubiquitin is a tag protein that marks another protein for degradation (Fig 1A). To tag ubiquitin to a substrate protein, ubiquitin is first activated by an ubiquitin-activating enzyme E1, followed by conjugation to an ubiquitin-conjugating enzyme E2, and then transfer to the protein substrate bound to an ubiquitin ligase E3, on either the α-NH_2_ of methionine or ε-NH_2_ of a lysine on the substrate. The ubiquitination cascade is repeated with ubiquitylation of the ubiquitin attached to the protein to produce polyubiquitinated proteins, which are recognized by ubiquitin receptors on the 19S regulatory particle of the proteasome and removed by deubiquitinases (Dubs) prior to unfolding by the ATPases at the base of the 19S. The unfolded proteins are then translocated into the 20S proteasome through the opened gate of the α ring and hydrolyzed in the proteolytic chamber of the 20S particle.

A homologous proteasomal degradation system has been identified in *Mycobacteria* (Fig 1B) [5,6]. In this system, prokaryotic ubiquitin-like protein (Pup) plays the role of ubiquitin in the UPS. Several homologous proteins of UPS have been identified. Dop is a dual-functional enzyme that activates Pup [7] and removes Pup from a pupylated protein [8,9]. PafA works as a ligase, catalyzing the coupling reaction of γ-carboxylate group of C-terminal glutamate of Pup-^C^Glu with an ε-amino group of a lysine residue of a protein substrate via an isopeptide bond, mediating proteasomal degradation [7,10]. Mpa and PafE were identified as an ATP-dependent proteasome activator and an ATP-independent activator, respectively [11–13].

There are 3 types of proteasomes expressed by human cells, the constitutive proteasome (c-20S) in all cells [14]; the immunoproteasome (i-20S) in immune cells or cells stimulated with interferon-γ or cells at inflammatory sites [15–20]; and the thymoproteasome (t-20S) in epithelial cells of the thymus cortex [21,22]. Hybrids of c-20S and i-20S also form. Within c-20S reside 2 copies of each of 3 proteases with distinct specificities, β1 (caspase-like), β2 (tryptic-like), and β5 (chymotryptic-like). In i-20S, β1, β2, and β5 are replaced by β1i, β2i, and β5i, respectively. Mice lacking i-20S subunits are generally immunocompetent, though they have enhanced susceptibility to coxsackievirus B3 [23], *Toxoplasma gondii* [24], and *Listeria monocytogenes* [25]. It is likely that the list of susceptible pathogens will grow. The thymoproteasome (t-20S) differs from the i-20S by replacing β5i with β5t (PSMB11), resulting in a composition of active β1iβ2iβ5t subunits [22]. The t-20S plays important roles in killer T cell development [22,26,27]. The proteasome not only controls many critical cellular checkpoints through degradation of inhibitors, but also generates peptides for antigen presentation [4,28]. Highly specific proteasome inhibitors markedly limit the overall supply of peptides for MHC class I molecules and thus block antigen presentation [29].

The proteasome is a clinically validated drug target for hematologic neoplasms, with 3 drugs approved by the FDA: Bortezomib, Carfilzomib, and Ixazomib [30]. Highly secretory cells, such as plasma cells and malignant multiple myeloma cells, are hypersensitive to the loss of proteasome functions, which is the rationale for the treatment of multiple myeloma and off-label use of such drugs in transplantation to prevent rejection. However, by the same token, proteasome inhibitors intended to target pathogenic microbes without sparing host proteasomes can be expected to be toxic and immunosuppressive.

**Fig 2:**
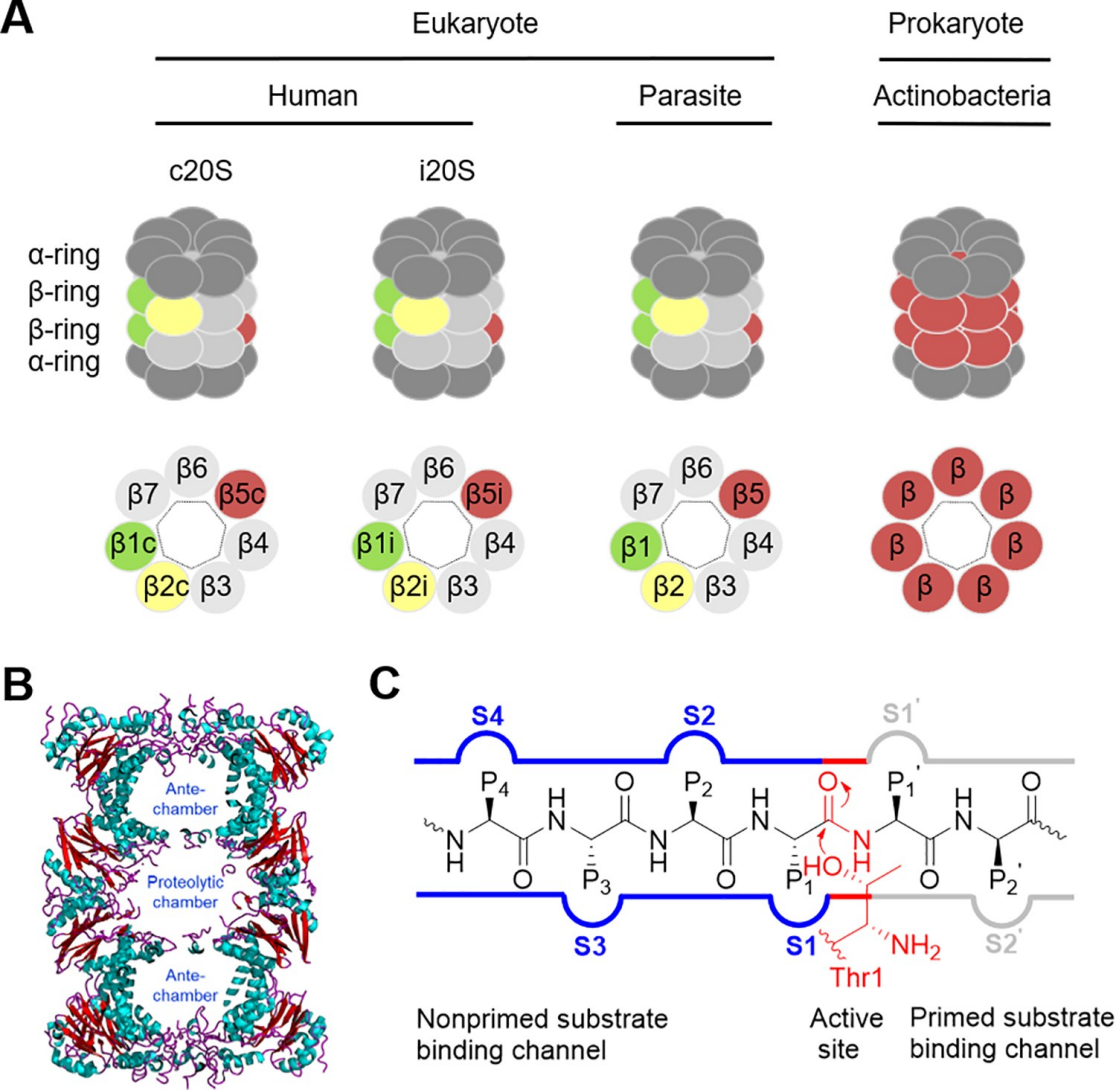
Illustration of 20S proteasomes of eukaryotes and prokaryotes. (A) 20S proteasomes are highly conserved in structure from bacteria to humans. Each 20S proteasome consists of 14 α subunits and 14 β subunits, organized as a barrel-shaped compartmentalized protease with α and β subunits in α_7_β_7_β_7_α_7_ order. Outer rings are composed of α subunits and inner rings of β subunits. Only β subunits are proteolytically active in prokaryote proteasomes, and only β1, β2, and β5 are proteolytically active in eukaryotic proteasomes. (B) Three chambers are formed in each 20S proteasome. The 2 outer chambers formed by α rings abutting the β rings are proposed to prevent unfolded protein substrates from refolding again prior to hydrolysis in the proteolytic chamber. (C) Substrate binding cleft located at the β subunits. The nucleophilic hydroxy group of the N-terminal Thr^1N^ at β of prokaryotic 20S proteasomes and β1, β2, and β5 of eukaryotic 20S proteasomes attacks the amide bond at the preferred site of a protein substrate.

The 20S proteasome core particle (CP) of eukaryotes and prokaryotes shares a similar architecture (Fig 2). The 20S CP is composed of 28 subunits coaxially stacked in 4 heptameric rings. The 2 inner rings, each consisting of 7 β subunits, are sandwiched between the 2 outer rings, each formed by 7 α subunits. Eukaryotic proteasomes contain 2 copies of 7 different α-subunits (α_1–7_) and 7 different β-subunits (β_1–7_). Only 3 subunits—β1, β2, and β5—are proteolytically active sites (Fig 2A). Bacterial proteasomes are simplified, with 1 or 2 α and 1 or 2 β subunits. In *Mycobacterium tuberculosis* (Mtb), there is 1 α and 1 β subunit, encoded by genes *prcA* and *prcB*, respectively. Therefore, this proteasome contains 14 active sites, 7 in each of the 2 β rings. Mycobacterial proteasome β subunits exhibit broad proteolytic activity [31].

All the proteolytic active subunits of eukaryotic and actinobacterial proteasome rely on the hydroxyl group of the N-terminal threonine (Thr^1N^) as the nucleophile, which attacks the carbonyl carbon of the scissile peptide bond to mediate the peptide bond cleavage. The nonprime and prime substrate-binding pockets are located at the N- and C-terminal sides of the scissile peptide bond, respectively (Fig 2C).

Below, we summarize recent progress in the development of proteasome inhibitors as antimicrobial agents.

### Eubacterial pathogens

#### Mycobacterium spp.

The only known bacterial pathogens with proteasomes are mycobacteria, including Mtb, *Mycobacterium leprae*, and nontuberculous *Mycobacteria* (NTM) such as *Mycobacterium abscessus* and *Mycobacterium avium*. Mtb is thought to have caused more deaths than any other bacterial pathogen during human history. The growing resistance of Mtb to anti-Mtb drugs coincides with the spread of immune-suppressing viral infections (HIV) and metabolic states (e.g., diabetes), posing a great challenge to global public health. Novel anti-Mtb drugs are urgently needed to combat tuberculosis.

**Fig 3:**
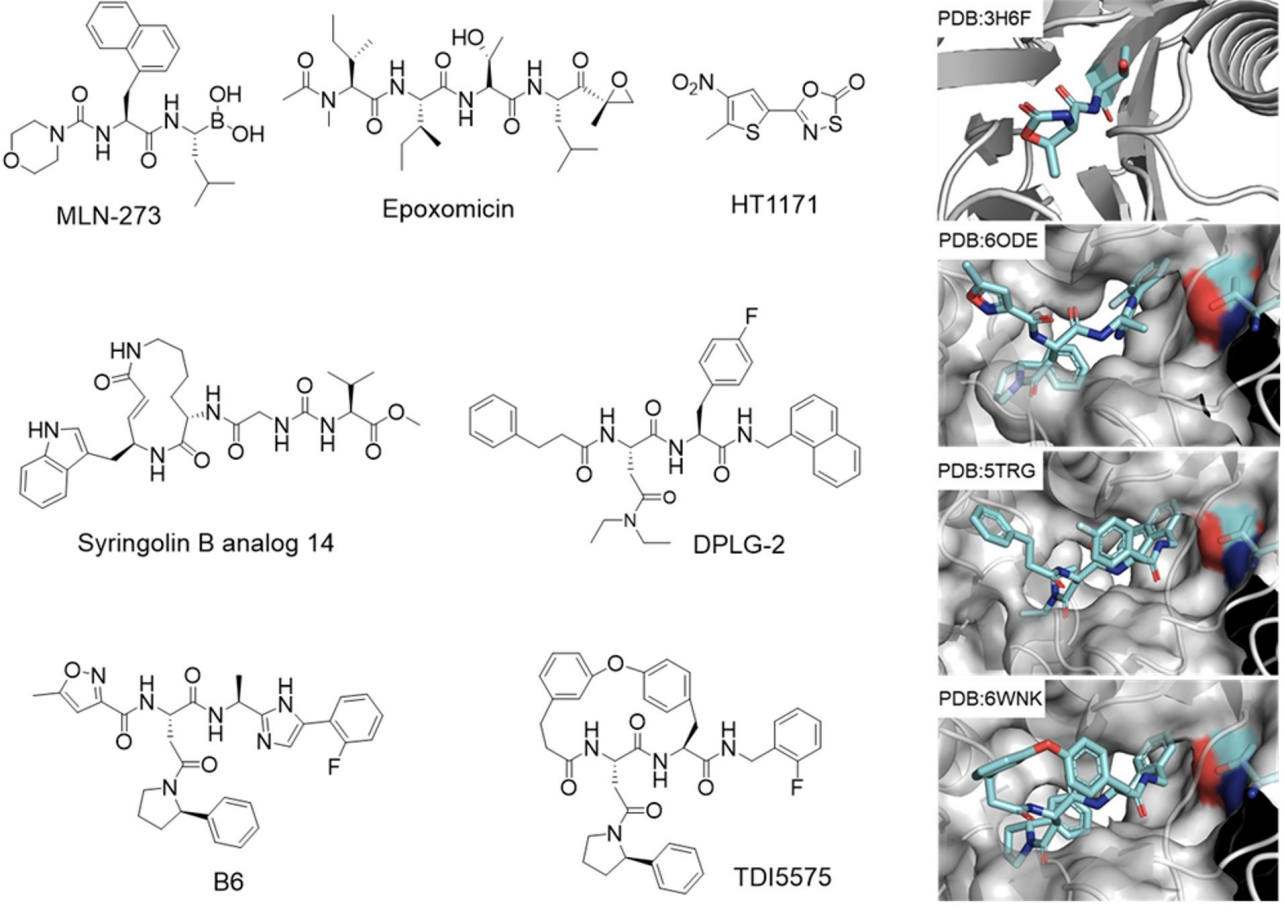
Proteasome inhibitors tested for species selectivity of inhibition of Mtb20S. MLN-273 and epoxomicin were first tested during the discovery of the role that the Mtb20S plays in the defense of Mtb against nitrosative stress. HT1171 and GL5 are irreversible inhibitors discovered in a high-throughput screening campaign. Syringolin B analog 14 is an irreversible inhibitor developed based on the natural product syringolin B. DPLG-2, B6, and TDI5575 are noncovalent reversible inhibitors that are highly selective for Mtb20S over human proteasomes.

A screen of 10,100 transposon mutants of Mtb identified enzymes whose disruption sensitized Mtb to oxidative/nitrosative injury [13]. The 12 mutants identified with unique transposon insertions represented 7 genes. Five of the mutants were disrupted in 2 genes annotated as serving the proteasome [13]. Two proteasome-specific inhibitors, a peptidyl boronate MLN-273 (Table 1) [32] and epoxomicin [33] (Fig 3), each were mycobactericidal during recovery of Mtb from exposure to reactive nitrogen intermediates (RNIs) [13]. One of the genes encodes an ATPase of the AAA family called Mpa [12]. The authors found that recombinant Mpa displayed Walker box-dependent ATPase activity and self-assembled into a hexamer closely resembling proteasome-associated ATPases in other species [12], and activated Mtb20S [34,35]. The second gene was termed *pafA* for proteasome accessory factor. This was later found to encode a ligase activity [10]. Recombinant Mtb *prcBA* (Mtb20S) genes were cloned and expressed and their encoded proteins characterized structurally and biochemically [31,36,37]. Deletion of N-terminal (2–9) of α-subunits (Mtb20OG) mimics a physiological mechanism for gate opening and has a higher specific activity than the wild type without a change in substrate preference [37]. Darwin and colleagues subsequently discovered a Pup [38]. Weber-Ban and colleagues discovered the Dop protein, which activates Pup by converting the C-terminal tripeptide of Pup (GGQ) to GGE [7]. Genetic studies found that deletion of *mpa*, *pafA*, *prcBA*, and *dop* each render Mtb highly susceptible to nitric oxide and unable to survive in lungs of mice [12,13,39,40], thus commending Mtb 20S as a target for anti-Mtb drug development.

**Table 1:** Inhibitory activity of Mtb20S inhibitors.

	IC_50_ (nM) or (k_obs_/I*, M^−1^s^−1^)			EC_50_ (nM)		Ref.
	Mtb20S	c-20S	i-20S	Anti-Mtb	HepG2	
MLN-273	1.6	0.15	NA	+	NA	[13,32]
GL5	376.4*	0.4*	NA	+	>100,000	[41,42]
HT1171	2,134*	10.1*	1012.2*	+	>100,000	[41,42]
Syringolin B analog 14	1,013*	13.6*	NA	+	NA	[43]
DPLG-2	15	70,000	54,700	+	>100,000	[44]
B6	8	>100,000	>100,000	−	>100,000	[45]
TDI5575	7.4	>100,000	>100,000	+	>100,000	[46]

NA, not available.

+: mycobactericidal against nonreplicating Mtb under NO stress; −: inactive.

### Mtb20S-selective inhibitors

#### Oxathiazole-2-ones

Oxathiazole-2-ones GL5 and HT1171 (Fig 3) were identified as first-in-class selective Mtb20S inhibitors through a high-throughput screening of 20,000 compounds for the hydrolysis of fluorogenic substrate Suc-LLVY-AMC by Mtb20S [41]. GL5 and HT1171 showed >1,000-fold selectivity against Mtb20S over human c-20S (Table 1). Oxathiazol-2-ones are competitive, irreversible, and mechanism-based inhibitors of Mtb20S but are substrates of the c-20S. Biochemical and structural analyses confirmed that oxathizol-2-ones cyclocarbonylate the OH and NH_2_ of Thr^1N^ in the active site of the Mtb20S. GL5 and HT1171 kill nonreplicating Mtb under NO stress. Crystal structures of an Mtb20SOG, in which the N-termini of a subunit following treatment with either inhibitor showed that the S4-H1 loop in Mtb20S underwent a marked conformational change that facilitated the cyclocarbonylation of the active site. However, this class of proteasome inhibitors inhibits i-20S, likely via a similar mechanism to that against Mtb20S [42].

#### Syringolin

Syringolin B analog 14 (Fig 3) was reported as a covalent, irreversible, and selective Mtb20S inhibitor. It was rationally designed by introducing P1 Trp and P3 Gly. Syringolin B analog 14 shows 74-fold selectivity against Mtb20S over c-20S and is active against Mtb under NO stress (Table 1) [43].

#### N,C-capped dipeptides

A library of 1,600 N,C-capped dipeptides with varying P1 (C-cap), P2, P3, and P4 (N-cap) was screened against Mtb20S [44]. The most species-selective compounds shared a fully substituted nitrogen atom at the P3 Asn side chain. DPLG2 inhibited Mtb20S in a time-dependent manner with *K*_i_ 15 nM and showed 4,667-fold selectivity over c-20S and 3,647-fold over i-20S (Fig 3 and Table 1). DPLG2 can penetrate mycobacteria and kill nonreplicating Mtb under nitrosative stress. Co-crystal structures of the Mtb20SOG with DPLG2 and 5 other analogs showed that the inhibitors bind to each of the 14 β subunits of Mtb20S in a short antiparallel β-strand form. Ser-20 and Gln-22 of Mtb20S, which are not conserved in human β5 and β5i, form hydrogen bonds with the dipeptides, which greatly contributes to the specificity for Mtb over the human homologs.

#### Phenylimidazole-based peptidomimetics

In order to improve the pharmacokinetic properties of DPLG2, the C-terminal amide was replaced with its bioisosteres phenylimidazole [45]. An iterative automated microfluidic system allowed efficient and rapid structure–activity relationship (SAR) studies to improve phenylimidazole-based proteasome inhibitor B6 (Fig 3 and Table 1), which showed potent inhibitory activity against Mtb20S (IC_50_ = 8 nM) and >12,500-fold selectivity over human c-20S and i-20S. It is surprising that phenylimidazole-based proteasome inhibitors are intrinsically selective for Mtb20S over c-20S and i-20S. The co-crystal X-ray structure of B6 and the Mtb20SOG showed that the imidazole ring forms 2 additional hydrogen bonds with Gly-47 and Ser-20, and modeling studies showed that the phenylimidazole moiety does not fit in to S1 pocket of either c-20S or i-20S, thus shedding light on the species selectivity of the phenylimidazole-based Mtb20S inhibitors.

#### Macrocyclic peptides

Macrocycle TDI5575 was developed using a strategy to covalently link the P2 and P4 phenyl groups of DPLG2 (Fig 3 and Table 1) [46]. S2 and S4 binding pockets are partially exposed to solvent. Cyclization provides rigidity that often improves potency and pharmacokinetic properties. The co-crystal structure of Mtb20SOG with TDI5575 showed that the bulky 2-phenyl substituent on the pyrrolidinyl of the P3-Asn binds to the shallow but wide S3 pocket, affording strong species selectivity. Treating Mtb with TDI5575 led to the accumulation of Pup-tagged GFP and to death of nonreplicating Mtb under NO stress [46].

### Parasitic pathogens

Neglected tropical diseases (NTDs) are a serious public health and economic burden in affected low- and middle-income countries. NTDs can be caused by bacteria, viruses, and parasites. The protozoa and helminths that cause NTDs, in addition to the *Plasmodia* that cause malaria, affect hundreds of millions of people. Research in drug development for NTDs and malaria has actively sought novel targets. The life cycles of both monoxenous and heteroxenous protozoal parasites require stage-specific transformation. The parasite proteasome plays an essential role of in these stage-specific transformations through highly regulated protein turnover, inhibition of which is detrimental to the viability and infectivity of the parasites.

### Protozoal parasites

#### Plasmodia

*Plasmodia* cause 214 million new cases of malaria each year and approximately 450,000 deaths. Most of those dying are children under 5 years of age [47]. The fast acting and potent artemisinins (ARTs) are key to successful antimalarial combination therapy [48,49]. However, ART resistance is now firmly established in the Greater Mekong Subregion (GMS) and complicated by the concomitant spread of resistance to important partner drugs [50–56]. A recent report of spread of clinically ART-resistant *Plasmodium falciparum* (Pf) in Africa with independently emerged mutations in Kelch13 protein from the GMS resistant parasites [57] is the testament of urgent needs to develop antimalarials with novel targets, particularly those that may interfere with mechanisms of ART resistance [58,59].

Early in the development of proteasome inhibitors, several studies validated the Pf proteasome (Pf20S) as a therapeutic target: (1) lactacystin blocked replication of the schizont stage of Pf in human erythrocytes in vitro and inhibited the infectivity of the sporozoite stage for human liver HepG2 cells [60]; (2) epoxomicin inhibits Pf development at ring and early trophozoites stages and causes accumulation of polyubiquinated proteins [61]; (3) bortezomib and ZL3B are active against Pf strains irrespective of whether the parasites are resistant to chloroquine or pyrimethamine [62]. However, none of these early proteasome inhibitors are species selective, and some are not sufficiently drug-like for in vivo efficacy studies.

**Fig 4:**
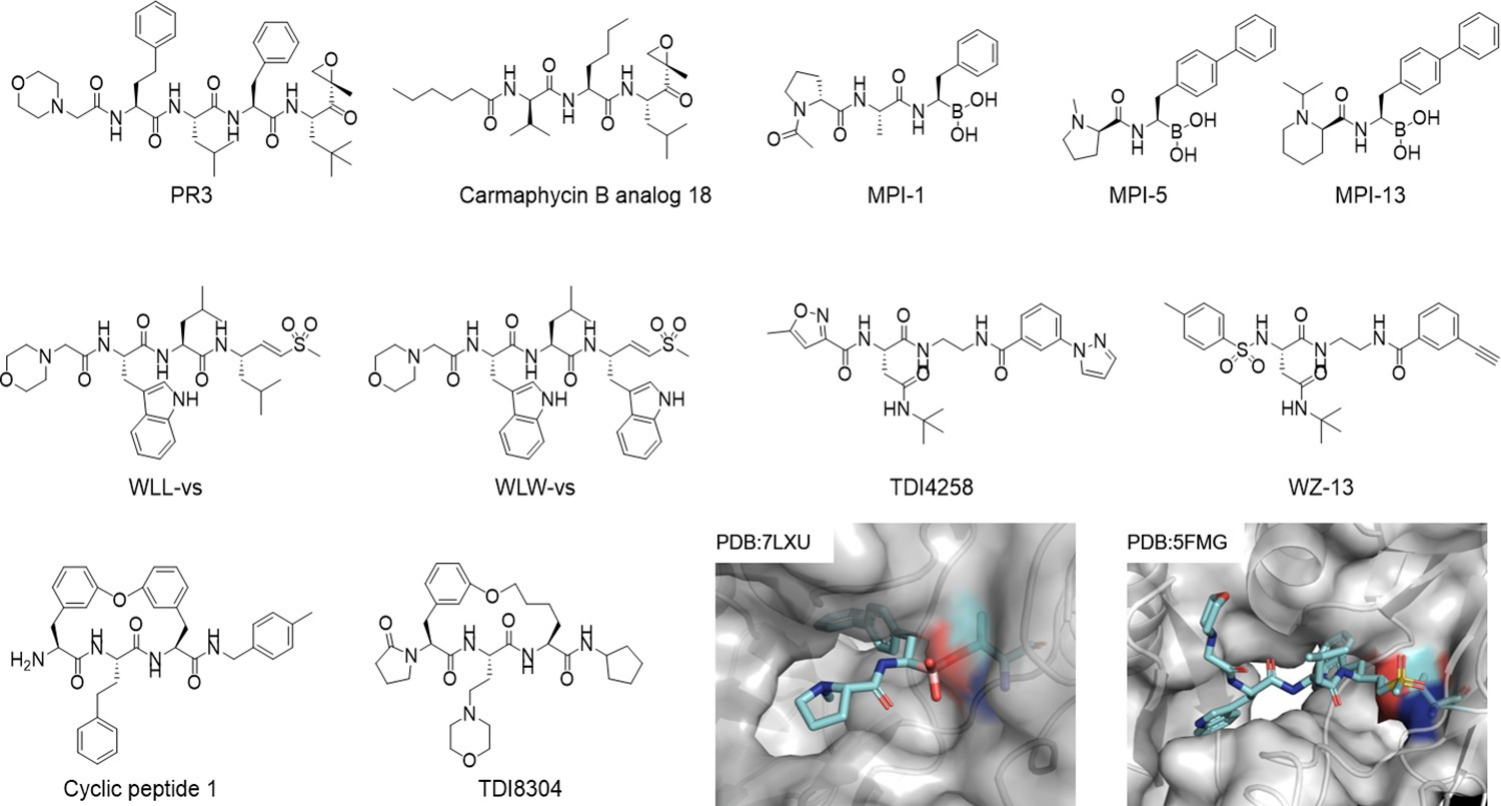
Pf20S-selective proteasome inhibitors. Inserted structures show binding of the MPI-5 to β5 (PDB: 7LXU) and binding of WLW-vs binds to β2 of the Pf20S (PDB: 5FMG).

Following the early validation of Pf20S as a therapeutic target, Li and colleagues screened a library of 670 analogs of carfilzomib and identified PR3 as potent against Pf and the first compound demonstrating in vivo efficacy in mice infected with *P*. *berghei* without apparent host toxicity (Fig 4 and Table 2) [63]. The markedly reduced host toxicity was attributed to its weak inhibition of c-20S β5c and no inhibition of c-20S β2c. Additionally, RP3 is a weak inhibitor of Pf20S β5, implying that Pf is hypersensitive to the loss of proteasome function. The same lab screened a 1,600 N,C-capped dipeptide library with purified Pf20S and identified macrocyclic compound 1 (Fig 4 and Table 2) with modest species selectivity at the enzyme level but superb selective cytotoxicity against Pf over nontransformed human foreskin fibroblast cells [64]. The enhanced antiparasite activity of the compound relative to its inhibition IC_50_ of Pf20S is likely attributable to its coinhibition of β5 and β2 of the Pf20S and to a high level of uptake and accumulation by the parasites. In another study, Li and colleagues evaluated the coinhibition of β5 and β2 of Pf20S with a human proteasome β2-selective inhibitor, LU101, in addition to the Pf20S β5-specific inhibitor PR709A [65]. They found that selectively inhibiting β5, but not β2, blocked parasite replication during blood stage schizogony, while inhibiting both β5 and β2 enhanced parasite killing at all blood stages of the parasite life cycle and reduced the parasite load to barely detectable levels in mice infected with *P*. *chabaudi*. The observation of a synergistic effect from coinhibition of β5 and β2 of Pf20S conforms with the observation of a synergistic effect of coinhibition of β5 and β2 of human proteasomes in cancer cells and may suggest that synergistic effects of coinhibition of β5 and β2 could pertain to other eukaryotic pathogens [66,67].

**Table 2:** Inhibitory activity of Pf20S inhibitors and antimalarial activity.

	IC_50_ or K_I_* (nM)			EC_50_ (nM)		Ref.
	Pf20S	c-20S	i-20S	Pf 3D7	HepG2	
PR3	94	310	NA	129 (60 hours)	>30,000 (HFF)	[63]
Cyclic peptide 1	1,250	9,220	>100,000	34.5	>100,000 (HFF)	[64]
WLW-vs	800 (β2)	8,000 (β5)	23 (β5i)^a^	290	12,800 (HFF)	[69]
WLL-vs	900 (β2) 800 (β5)	1,000 (β5)	NA	6.2	1,530 (HFF)	[69]
Carmaphycin B analog 18	371*	39,213*	NA	3.27	1,240	[72]
TDI-4258	58.5	10,110	780	29	>100,000	[73]
WZ-13	4.7	430	112	3.1	>11,000	[75]
TDI-8304	60	81,800	>100,000	9	>100,000	[76]
MPI-1	1,500	220	28	120	6,700	[77]
MPI-5	5	85	24	21	2,620	[78]
MPI-13	12	230	24	11	930	[78]

HFF, human foreskin fibroblasts; NA, not available.

^a^Ref. [73].

Tschan and colleagues investigated a novel class of proteasome inhibitors with a sulfonyl fluoride (VF) warhead [68]. The authors identified PW28 (Cbz-LLLL-VF) as highly active against Pf 3D7, D10 (atovaquone-resistant), and Dd2 (multidrug-resistant) at different asexual stages. PW28 inhibited gametocyte maturation without cytotoxicity against HeLa and HEK293 T cells even at 500 μM. PW28 targeted both Pf20S β5 and β2 in an activity-based probe assay, but no quantification of the inhibition was reported. Although PW28 showed modest in vivo activity in mice infected with *P*. *berghei*, more than half of the mice treated with PW28 died, suggesting that PW28 has host targets. Given the reactivity of the sulfonyl fluoride with nucleophilic side chains of serine, threonine, tyrosine, and lysine, it is critical to improve the selectivity between human and parasite proteasomes, as well as to avoid or reduce reactivity with other host proteins.

The need to optimize Pf20S inhibitors called for the better understanding of substrate profiling and the structure of Pf20S for improving potency, selectivity, and pharmacokinetic properties. Bogyo and colleagues used a set of 228 diverse synthetic tetradecapeptides to investigate the amino acids at and near the fission site preferred by Pf20S and not Hu 20S. The authors found that tryptophan at both P3 and P1 in either substrate or inhibitor offered much improved selectivity between Pf20S and hu 20S, compared to the other compounds tested. They further solved the first Pf20S cryo-EM structure with a β2 specific inhibitor WLW-vs at 3.6 Å (Fig 4 and Table 2) [69]. However, substrate profiling with the synthetic oligopeptides provided an overall picture of amino acid preferences at binding pockets without distinguishing among the different active subunits. For example, WLW-vs is highly selective for Pf20S β2 over hu c-20S but potently inhibits human i-20S β5i. Changing P1 tryptophan of WLW-vs to leucine results in WLL-vs. WLL-vs inhibits both β2 and β5 of Pf20S but also inhibits β5 of hu 20S, and not β2, potentially enhancing selectivity toxicity for *Plasmodium* over human cells (Fig 4 and Table 2). The irreversible mechanism of the proteasome inhibitor supported testing a single bolus dose of 35 mg/kg in mice infected with *P*. *chabaudi*, which resulted in elimination of parasitemia without apparent toxicity to the host mice. However, it remains a challenge to develop peptide vinyl sulfones as drugs because the warhead vinyl sulfone irreversibly reacts not only with the nucleophilic hydroxyl group of the active site threonine of proteasomes but also the sulfhydryl group of the active site cysteine of cysteine proteases [70]. More research is needed to improve both the drug-likeness and specificity of the peptide vinyl sulfones.

In order to improve species selectivity of peptide-based epoxyketone proteasome inhibitors, Gerwick and colleagues started with the natural product carmaphycin B, a tripeptide α,β-epoxyketone, with potent sexual and asexual antimalarial activity and significant cytotoxicity against human cell lines [71,72]. The authors systemically investigated carmaphycin B analogs with various P2 and P3 moieties and monitored the SAR by in vitro antimalarial activity and host cytotoxicity. Replacing the P2 methionine sulfone and P3 L-valine of carmaphycin B with norleucine and D-valine respectively resulted in analog 18 (Fig 4 and Table 2), which retained potent antimalarial activity against asexual blood stages and gametocytes with reduced host cytotoxicity and a 100-fold wider therapeutic window than the parent compound.

The benefit of having a covalent warhead in a proteasome inhibitor is to improve the on-target residence time and affinity. However, the use of covalent warhead poses a challenge for optimizing selectivity for microbial proteasomes over human proteasomes, particularly for Pf 20S, which has a similar substrate preference profile to that of human i-20S. To develop Pf20S inhibitors that are selective over both c-20S and i-20S, we screened a library of noncovalent proteasome inhibitors against a Pf lysate with suc-LLVY-AMC as substrate and identified an asparagine-ethylenediamine (AsnEDA) class of proteasome inhibitors that inhibited suc-LLVY-AMC hydrolyzing activity [73]. AsnEDAs are β5 inhibitors [74]. Hit optimization yielded a potent antimalarial, TDI4258, with modest selectivity over c-20S (172-fold) and i-20S (13-fold) (Fig 4 and Table 2). Synergy was demonstrated between dihydroartemisinin (DHA) and TDI4258, between DHA and TDI4258 and WLW-vs, and between TDI4258 and WLW-vs [73]. Analog PKS21224 has a moderate parasite kill rate and PKS21004 was effective against *P*. *berghei* in preerythrocytic stages. SAR studies improved the potency and selectivity of this class of compounds (WZ-13) (Fig 4 and Table 2); however, despite much effort in optimizing PK properties, microsomal stability remained a liability [75].

Inspired by the macrocyclic compound 1 [64], we conducted an extensive SAR study to improve the pharmacokinetic properties of this scaffold. We focused on substitution at (1) P5, to reduce hydrogen bond donor count and improve passive permeability; (2) P1, P3, and the tether, to improve solubility and microsomal stability; and (3) P3, to reduce LogD by replacing the phenyl with a morpholino group. In addition, (4) we further modified the P1 moiety to reduce a lability to hydrolysis. The hit to lead optimization yielded TDI-8304, which is highly potent against Pf 3D7 and has marked selectivity for inhibition of Pf20S over both c-20S and i-20S (Fig 4 and Table 2). TDI-8304 demonstrates specific, time-dependent inhibition of the β5 subunit of the Pf20S with a fast parasite kill rate and has favorable pharmacokinetic properties. This compound kills ART-sensitive and ART-resistant Pf isolates in vitro and markedly reduced parasitemia in humanized, Pf-infected mice when given at 50 mg/kg by subcutaneous injection twice a day for 4 days [76].

Peptide boronates are the pillar of clinically used proteasome inhibitors. The strong B-O bond (809 kJ/mol for bond dissociation energy) ensures that even a short peptide or even one amino acid possesses potent inhibitory activity, in contrast to other peptide-based proteasome inhibitors. For example, tripeptides are generally needed to confer potency on epoxyketone or vinyl sulfone warheads. Boron compounds can also undergo easy interconversion between neutral trigonal planar *sp*^*2*^ and tetrahedral *sp*^*3*^ hybridization states that greatly facilitate the target engagement. Another advantage of peptide boronates for proteasome inhibitors is that they form a covalent and slowly reversible bond. This enhances residence time of the drug without completely shutting down the proteasome function, which would be detrimental to the viability of the cells. Building on earlier work of Reynolds with peptide boronate proteasome inhibitors [62], Tilley and colleagues, in a collaboration with a team at Takeda Pharmaceutical company led by Lawrence Dick, screened a peptide boronate library for Pf20S inhibition and selected 4 compounds for kinetic and biochemical analysis [77]. MPI-4 was not as selective as other reported proteasome inhibitors but was highly potent against Pf20S β5 and β1 and has t1/2 approximately 65 minutes on Pf20S β5, indicating a tight binding mechanism (Fig 4 and Table 2). However, the in vivo antimalaria activity of these compounds appeared to be markedly affected by their limited selectivity and corresponding potential to bind the host constitutive proteasome. For example, MPI-4 has an IC_50_ of 10 nM for Pf20S β5, 1.4 nM for c-20S β5c, and EC_50_ 61 nM against Pf3D7, whereas MPI-1 has an IC_50_ of 1500 nM for Pf20S β5 and 220 nM for c-20S β5c, yet MPI-1 is only 2-fold less potent than MPI-4, with EC_50_ 120 nM against Pf3D7. This observation may suggest that the highly abundant c-20S in red blood cells act as a sink for proteasome inhibitors that bind c-20S. The consortium continued their optimization of boronate analogs to improve the selectivity and antimalarial potency. They designed, synthesized, and evaluated a series of amino-amide boronates containing a single amide bond in the backbone [78]. The authors found that replacing the P1 phenyl group of MPI-1 with biphenyl group was able to retain the potency and improve the selectivity for Pf20S over c-20S. For example, MPI-5 and MPI-13 with different N-caps showed IC_50_ 5 nM and 12 nM against Pf20S β5, and 17- and 19-fold selectivity over c-20S β5c, respectively (Fig 4 and Table 2). MPI-5 and MPI-13 also displayed significantly improved oral bioavailability to 50% and 78%, respectively. The authors solved a cryo-EM structure of Pf20S with MPI-5 at 3.4 Å and found that MPI-5 only bound to the β5 active subunit, not to the β1 nor β2 active sites. MPI-13 reduced the parasitemia below the limit of detection when given to the mice infected with Pf at 25 mg/kg *p*. *o*. once daily for 4 days [78]. However, the lead compound MPI-13 still possesses potent inhibitory activity against i-20S β5i with IC_50_ 24 nM, which will likely compromise the host immunity against the parasite as it has been shown that impaired immunoproteasome function exacerbate the parasite, viral, and bacterial infections [23,79,80], thus more work is needed to improve the selectivity of proteasome inhibitor. Boronate pharmacophores are increasingly found in clinical drugs, improving the species selectivity of peptide-boronates for Pf20S would likely be advantageous in drug development.

#### Leishmania and Trypanosoma

Three important kinetoplastid infectious diseases are human African trypanosomiasis (also known as sleeping sickness), Chagas disease, and leishmaniasis, which mainly affect people in Africa, Latin America, the Middle East, and Asia [81]. Sleeping sickness and Chagas disease are caused by the parasites *Trypanosoma brucei* (Tb) and *Trypanosoma cruzi* (Tc), respectively. Leishmaniasis, caused by more than 20 different species of *Leishmania*, has 3 main clinical forms—cutaneous, mucocutaneous, and visceral leishmaniasis (VL). VL is the most severe form and is caused by *Leishmania donovani* (Ld) and *Leishmania infantum* (Li). Drugs to treat these infections are few and have shortcomings including high toxicity, prolonged treatment duration, high cost, and difficult administration [82]. New oral treatments for kinetoplastid diseases are urgently needed. Given the genomic conservation among these kinetoplastids [83], a drug targeting a conserved target could potentially treat all 3 diseases.

**Fig 5:**
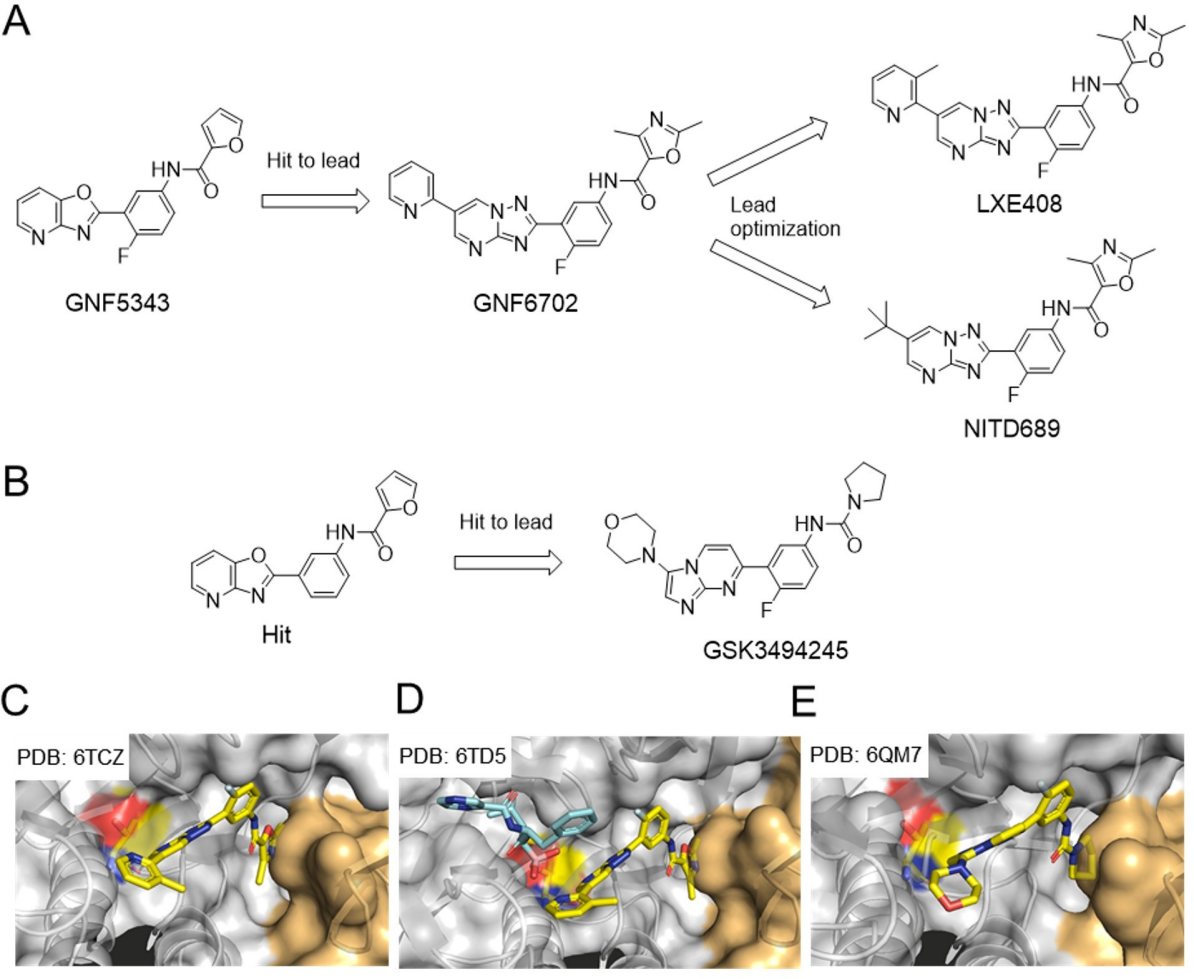
**Development of preclinical candidates LXE408 (A) and GSK3494245 (B), which selectively targets the kinetoplastid proteasomes.** Cryo-EM structures show that LXE408 (**C**; PDB: 6TCZ) and GSK3494245 (**D**; PDB: 6QM7) bind to the intersection of β4 (orange) and β5 (white) of the *Leishmania tarentolae* 20S, respectively [85,86]. Cryo-EM structure of *L*. *tarentolae* 20S with both bortezomib (cyan) and LXE408 (yellow) is shown in **E** (PDB: 6TD5) [85]. Active site Thr is shown in yellow.

A library of 3 million compounds was screened by Genomics Institute of the Novartis Research Foundation (GNF) in a proliferation assay on Ld and Tb [84]. Beginning with the hit GNF5343, a hit to lead optimization campaign led to the design and synthesis of 3,000 compounds to improve bioavailability and potency against intramacrophage Ld. Replacing the azabenzoxazole center with a triazolopyrimidine core improved the potency against intramacrophage Ld, whereas replacing the furan moiety with a dimethyloxazole group reduced the toxicity. Replacing the chlorine atom with a fluorine improved the cytotoxicity profile over mammalian cells. SAR studies yielded a lead compound, GNF6702, which showed low clearance (2.0 ml/min/kg), acceptable bioavailability (34%), and 400-fold improvement in potency against intramacrophage Ld compared to the hit (Fig 5A).

In a mouse of model of VL, twice-daily oral dosing 10 mg/kg GNF6702 for 8 days resulted in a 3-log_10_ decrease in parasite load in mice infected with Ld [84]. In a mouse model of cutaneous leishmaniasis (CL), a 5-fold reduction of parasite footpad burden was observed in mice infected with *Leishmania major* after treatment with 10 mg/kg GNF6702 twice daily for 10 days. In a mouse model of Chagas disease, GNF6702 eliminated the detectable parasite burden, matching the efficacy of benznidazole, an FDA-approved drug for Chagas disease. GNF6702 was further evaluated in a mouse model of human African trypanosomiasis. Once-daily dosing with 100 mg/kg GNF6702 reduced the parasite burden in the brain.

Evolution of resistance to GNF6702 and analogs in *T*. *cruzi* epimastigotes identified 2 clones with a I29M or F24L mutation in the proteasome β4 subunit that exhibited a 40-fold reduced susceptibility to GNF 6702. In contrast, the 2 mutant lines were not resistant to the proteasome inhibitors bortezomib and MG132. Tb overexpressing the proteasome β4^F24L^ subunit exhibited 70-fold increased resistance to GNF6702. Additionally, treating Tc epimastigotes with GNF6702 resulted in accumulation of polyubiquitylated proteins. These results suggested that GNF 6702 inhibits Tc by targeting the Tc proteasome. In agreement, GNF6702 selectively inhibited the kinetoplastid proteasome chymotrypsin-like activity over human proteasomes and showed a noncompetitive mode of inhibition [84].

The lead compound GNF6702 still suffered from limited oral absorption likely associated with low solubility. High crystal packing energy of a planar molecule like GNF6702 might account for poor solubility. Introducing a methyl group to the pyridine 3-position of GNF6702 yielded compound LXE408, which showed improved solubility and oral exposure (Fig 5A and Table 3) [85]. The cryo-EM structures of *Leishmania tarentolae* 20S with LXE408 shows that LXE408 binds at the prime site of the active site, between β5 and β4 subunits (Fig 5C). The authors also solved the cryo-EM structure of the *L*. *tarentolae* 20S with both bortezomib and LXE408, which clearly show that bortezomib and LXE408 flank the nonprime and prime sites of the active site Thr^1N^, respectively (Fig 5E) [85].

**Table 3:** Inhibitory activity and antiparasite activity of kinetoplastid proteasome inhibitors.

	IC_50_ (nM)		EC_50_ (nM)		F (%)	Ref.
	Ld20S	hu c-20S	*Leishmania donovani*	3T3		
GNF6702	35	>10,000	18	>20,000	34	[84]
LXE408	40	NA	40	NA	46	[85]
GSK3494245	160	13,000	1,600	>50,000 (THP-1)	18	[86]

F (%): oral bioavailability.

Ld20S, *Leishmania donovani* 20S; NA, not available.

A hit compound with a structure similar to that of GNF5343 was independently discovered from a phenotypic screen against *T*. *cruzi* by scientists at GlaxoSmithKline and the University of Dundee (Fig 5B) [86,87]. When initial medicinal chemistry efforts failed to balance potency, metabolic stability, and solubility, the investigators embarked on a scaffold hopping strategy. Replacing the bicyclic core with “reversed” scaffold, imidazo[1,2-a]pyrimidine, yielded compound GSK3494245, which showed slightly decreased potency against intramacrophage Ld (EC_50_ 1.6 μM), but much improved solubility (180 μg/mL), good in vitro metabolic stability (CL_int_ 0.8 mL/min/g), and good selectivity over mammalian cells (Table 3). Compound GSK3494245 maintained ex vivo potency against clinical *L*. *donovani* strains from East Africa and India. Twice-daily oral dosing with 25 mg/kg GSK3494245 for 10 days resulted in a >95% reduction of parasite burdens in a mouse model of VL infection. No noteworthy adverse effects were observed in a rat 7-day toxicology study at doses up to 300 mg/kg [86,87].

In order to determine the mechanism of action of the lead compound, a whole-genome Tb RNA interference (RNAi) RNA library was screened and pointed to the ubiquitin-proteasome system as the target of GSK3494245. Resistant Ld clones were generated and targeted DNA sequencing revealed mutations within the genes encoding β4 (T30A) and β5 (G197C or G197S) subunits of the parasite proteasome. An engineered Ld overexpressing β5G197C showed decreased susceptibility to the series of compounds, confirming the parasite proteasome as the target. Biochemical assays demonstrated that compound GSK3494245 selectively inhibited the chymotrypsin-like activity of the parasite proteasome. The cryo-EM structure of GSK3494245 with *L*. *tarentolae* proteasome was solved at 2.8 Å and revealed that GSK3494245 binds to a site that lies between the β4 and β5 subunits (Fig 5D).

### Helminths

#### Cestodes

Cestoda, such as *Echinococcus granulosus*, *Echinococcus multilocularis*, and *Taenia solium*, are important human pathogens that cause neglected diseases. In total, they account for around 22 to 55 million disability-adjusted life years (DALYs) and thereby are comparable to malaria, the leading parasitic disease (39 million DALYs) [88]. Surgical removal of a cyst is a preferred treatment, but if surgery is nonoperational, praziquantel and albendazole are often used. However, these drugs were not explicitly developed for cestode infections, hence not optimal. A screening of 426 FDA-approved drugs sought in vitro activity against *E*. *multilocularis* metacestodes and identified bortezomib as a potent antimetacestodal agent with an EC_50_ of 0.6 μM [89]. However, bortezomib showed limited in vivo efficacy in mice infected with *E*. *multilocularis*, likely due to the dose-limiting toxicity of bortezomib. For rational design of cestodal proteasome selective inhibitors, a systemic biochemical profiling of cestodal proteasomes will be necessary to identify sweet spots that could offer species selectivity.

### Schistosomiasis

**Fig 6:**
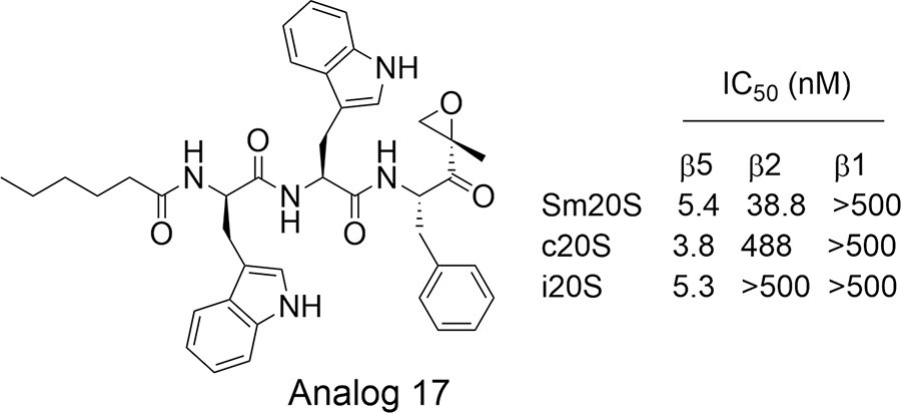
Inhibitor of Sm20S.

Schistosomiasis, caused by the *Schistosoma* blood fluke, affects 200 million worldwide. Treatment relies on one drug, praziquantel. Despite being an oral single-dose drug with few side effects, praziquantel is highly effective against adult *Schistosoma* worms but has limited efficacy against developing schistosomula and juvenile worms [90]. O’Donoghue and colleagues recently enriched *Schistosoma mansoni* proteasome (Sm20S) and confirmed that it possesses 3 typical proteasomal activities [91]. They showed that inhibition of Sm20S activity correlates well with inhibition of worm motility. In a screening of 11 peptide-based epoxyketone proteasome inhibitors against Sm20S, the authors identified a peptide epoxyketone analog 17 of carmaphycin B (Fig 6) with improved species selectivity at β2 and β1 active subunits and reduced HepG2 cytotoxicity [91]. However, the species selectivity for inhibition of β5 needs optimization.

### Perspective

Development of proteasome inhibitors as drugs has been challenging because of the essential role proteasomes play in many cellular functions. How to reduce toxicities from unwanted inhibition of host proteasomes has been gradually achieved with compounds directed against the proteasomes of several major infectious agents, such as Mtb, Pf, *Trypanosoma*, and *Leishmania*.

*Cryptosporidium parvum* is a protozoal parasite that causes cryptosporidiosis, a severe gastrointestinal disease. It is one of the most common causes of childhood diarrhea worldwide. Infection with *C*. *parvum* can have prolonged detrimental effects on the development of children and is life threatening in the setting of immunosuppression, such as in people with HIV/AIDS and in transplant recipients. Transcriptome studies established that components of UPS are highly expressed during the environmental survival and stresses in *C*. *parvum* oocysts [92] and stage transition from sporozoites to trophozoites [93]. As *C*. *parvum* lacks genes for nutrient synthesis and oocysts become less infectious with age [94], it is likely that the parasites heavily rely on their UPS to recycle amino acids by degrading nonessential proteins as well as by drawing on its reserves of amylopectin to survive shifts in environmental conditions. It remains to learn how proteasome inhibition will impact the biology and pathogenic potential of *Cryptosporidium*.

Another potential target for research in proteasome inhibitor development for infectious diseases are NTMs. Treatment options for NTM diseases are limited and can require years of chemotherapy with multiple antibiotics. It is not unusual that treatment fails. There is an urgent medical need for new antibiotics against the NTMs. NTMs have *prcB* genes, and it is possible that some of the Mtb20S-selective inhibitors under development will inhibit NTM proteasomes. However, the function of the NTM proteasomes remains to be characterized.

Another potential use of anti-infectious proteasome inhibitor drugs is for veterinary use, particularly to treat parasitic infections of livestock. For example, African animal trypanosomiasis causes serious economic losses [95–98]. Developing proteasome inhibitor drugs that are effective for trypanosomiasis in people and livestock would be a boon, given that the species of trypanosomes infecting people are not the same as those infecting livestock, so emergence of resistance in trypanosomes infecting one host population should not reduce efficacy of the drug in the other host population.

So far, characterization of species selectivity of proteasome inhibitors for microbes are limited to tests against c-20S and i-20S. To our knowledge, there has been no report of testing microbial proteasome inhibitors against human t-20S. Biochemical studies of t-20S are minimal. Florea and colleagues used a panel of activity-based proteasome probes to conclude that the amino acid preference of t-20S β5t is different from that of β5c and β5i, with a bias toward hydrophilic side chains of peptide based inhibitors [99]. Selectivity over t-20S will likely be very important when microbial proteasome inhibitor drugs are developed for infections in children, as inhibition of t-20S could stunt the development of adaptive immunity. Better understanding of the biochemistry of the t-20S will help in the development of microbial proteasome inhibitor drugs.

In sum, proteasome inhibitor drugs for infectious diseases are on the verge of benefiting patients. Additionally, small molecule probes and proteasome inhibitor libraries of various modalities have been established and are available to explore the role of proteasomes in other pathogens.
